# Differential associations of regional cerebellar volume with gait speed and working memory

**DOI:** 10.1038/s41598-022-06180-0

**Published:** 2022-02-11

**Authors:** Junyeon Won, Daniel D. Callow, Jeremy J. Purcell, J. Carson Smith

**Affiliations:** 1 Department of Kinesiology, School of Public Health, University of Maryland, 2351 SPH Bldg #255, College Park, MD 20742 USA; 2 Program in Neuroscience and Cognitive Science, University of Maryland, College Park, MD 20740 USA; 3 Maryland Neuroimaging Center, University of Maryland, College Park, MD 20740 USA

**Keywords:** Neuroscience, Physiology, Neurology

## Abstract

The relationship between gait speed and working memory is well-understood in older adults. However, it remains to be determined whether this relationship also exists in younger adults; and there is little known regarding the possible neural mechanism underlying the association between gait speed and working memory. The aims of this study are to determine if there is: (1) an association between gait speed and working memory performance; and (2) a mediating role of cerebellar subregion volume in the correlation between gait speed and working memory in healthy younger adults. 1054 younger adults (28.7 ± 3.6 years) from the Human Connectome Project were included in the analyses. A four-meter gait test was used to assess gait speed. The 2-back task was used to measure working memory performance [accuracy and response time (RT)]. T1-weighted structural MRI data (obtained using Siemens 3 T MRI scanner) was used to assess cerebellar subregion volumes. Linear regression and mediation analysis were used to examine the relationships between the variables after controlling for age, sex, and education. There was no association between gait speed and 2-back working memory performance in younger adults. Greater Crus I and whole cerebellar volumes were associated with better 2-back working memory accuracy. Greater VIIIa volume was associated with faster gait speed. Greater Crus 1 and VIIIa volumes were also associated with higher fluid cognition. The present study suggests that specific subregions of the cerebellar volumes are distinctively associated with gait speed and working memory performance in healthy younger adults.

## Introduction

Gait refers to the cyclic nature of an individual’s walking behavior and their unique movement pattern, and gait speed reflects an integration of the musculoskeletal, visual, and peripheral nervous systems^1^. Thus, assessing gait speed is a simple and reliable way of estimating functional capacity and motor function^2^. Beyond musculoskeletal mechanisms, gait control involves complex brain processes such as the integration of motor, perceptual, and cognitive functions (e.g., memory, attention, and executive function)^3,4^. Thus, it has been postulated that slowing gait may be a predictor for cognitive decline. Indeed, a body of longitudinal and cross-sectional evidence in older adults suggests an association between slower gait speed and lower global cognition^5,6^, attention and psychomotor speed^7^, verbal memory^8^, executive function^8,9^, working memory^10^, processing speed^11^, and greater risk of developing dementia^12^. However, since the relationship between gait speed and cognitive function has been primarily focused in geriatric and dementia studies, the evidence regarding gait speed, cognitive function, and cerebellum in younger adults is scarce. Characterizing this relationship in younger adults will provide a complementary and foundational understanding about the relationship between gait speed and working memory.

Since a report depicting impaired gait in World War I victims with gunshot-induced cerebellum damage^13^, it has been increasingly recognized that the cerebellum is an essential brain region for control and integration of motor activity^14^. Through feedforward and feedback loops, the cerebellum receives inputs from cerebral cortex and pontine nuclei and sends outputs to the thalamus and red nuclei^15^. The cerebellum integrates the inputs to fine-tune motor activity and regulates voluntary movements (e.g., posture and balance), visually guided movements, and balanced muscular activity. The cerebellum also identifies and regulates mismatch between actual and intended bodily movements (i.e., error correction)^16,17^. Cerebellum lesions are associated with gait dysfunction^18^ and greater cerebellar gray matter volume is related to faster gait speed^19,20^.

The cerebellum also engages in a broad range of cognitive processes, well-beyond its historical association with motor control and motor coordination. Cerebellar lesion studies reveal impaired executive function, spatial attention, planning, language processes, and learning and memory^21–23^. An anatomical foundation for the cerebellar contribution to cognition is the connection between the cerebellum and prefrontal and parietal cortices^24,25^. Through this fronto-parietal-cerebellar network, the cerebellum plays an important role in cognitive function^26^. Particularly, evidence regarding a cerebellar contribution to working memory in humans has been established based on human functional imaging studies. For example, there is a strong positive correlation between cerebellar gray matter volume and working memory performance^27^. In addition, greater cerebellar subregion gray matter volumes (e.g., lobules VI, right VIIIa, and Crus I) predicts better working memory performance^28,29^. Moreover, functional magnetic resonance imaging (MRI) activation within the bilateral regions lobules VI, Crus I, and VIIIa were observed during the n-back working memory task^30,31^.

Collectively, the literature provides evidence for the cerebellum’s involvement in both gait speed and working memory. Both gait speed and working memory also depend on information-processing ability and attention, which are primarily prefrontal-cortex dependent functions^32^. It is speculated that signals from the prefrontal regions may communicate via the fronto-cerebellar network and reach cerebellum to induce motor and cognitive outputs^19,33^. As both gait speed and working memory depend on information-processing ability and attention, which are primarily prefrontal-cortex dependent functions, the cerebellum may engage in both motor and cognitive functions via the fronto-cerebellar pathway. Given this, we hypothesized that certain cerebellar subregions may engage in both motor and cognitive functions even though each of the lobules has separate anatomical thalamocortical pathways that subserve different behaviors. A mediating role of the cerebellum would add important insights into our understanding of the shared neural mechanism underlying any potential associations between gait speed and working memory. To test this research question, we selected cerebellar subregions of interest that are linked to both gait speed and working memory. Working memory paradigms have been shown to elicit fMRI activity in the bilateral VIIb and right VIIIa^30,34^. The VIIb and VIIIa volumes have been associated with motor control and gait speed^35,36^. We also selected the Crus I, a subregion associated with attention and working memory^27^, and lobule V, a subregion involved in sensorimotor function including gait speed^19,36^. Therefore, we selected the cerebellar lobules VIIb, right VIIIa, V, and Crus I as volumetric regions of interest for the present study. We also quantified bilateral cerebellum volume to test whether the hypothesized mechanism is driven by total cerebellar volume rather than specific subregion volumes.

The aims of this study were to investigate the associations between (1) gait speed and cerebellar subregion volumes; (2) gait speed and working memory performance; (3) cerebellar subregion volumes and working memory performance; and (4) the possible mediating role of cerebellar subregions in the relationship between gait speed and working memory performance. We hypothesized that (1) faster gait speed would be associated with greater cerebellar subregion volumes; (2) faster gait speed would be associated with better working memory performance; (3) greater cerebellar subregion volumes would be associated with better working memory performance; and (4) greater cerebellum subregion volume would mediate the relationship between faster gait speed and better working memory performance. To test these hypotheses, we leveraged the publicly available cross-sectional dataset of healthy younger adults from the Human Connectome Project^37^. In a sizable and well-characterized younger sample, available data from Human Connectome Project including gait speed, working memory performance, and high-resolution MRI data were analyzed. As an index of working memory performance, we used n-back task performance. In addition to working memory performance, we also tested the fluid cognition composite score to test the specificity of the relationship between gait speed, cerebellar subregion volumes, and working memory. We also examined the association between bilateral thalamus volume, gait speed and working memory performance to test if the present findings are specifically linked to the cerebellum or if they could be linked to cerebellar thalamocortical pathways that contribute to voluntary movement^38^. Moreover, we tested the precentral gyrus, where the primary motor cortex is located, as a control region.

## Methods

### Participants

The publicly available younger adults data from University of Minnesota (Wu-Min) Human Connectome Project (https://www.humanconnectome.org/study/hcp-young-adult/document/1200-subjects-data-release) were used for the present study^37^. Healthy younger adults (19–35 years) were recruited from Missouri, using the recruiting strategy that reflected the population distribution of the US. Participants were excluded from the study if they had a history of psychiatric disorder, substance abuse, neurological, or cardiovascular disease, two or more seizures after age five or a diagnosis of epilepsy, any genetic disorder (e.g., cystic fibrosis or sickle cell disease), multiple sclerosis, cerebral palsy, brain tumor or stroke, premature birth, currently on chemotherapy or immunomodulatory agents, or history of radiation or chemotherapy that could affect the brain, thyroid hormone treatment in the past month, treatment for diabetes in the past month (other than gestational or diet-controlled diabetes), use of daily prescription medications for migraines in the past month, < 25 on the Mini Mental State Exam^39^ on the Day 1 visit, pregnancy, MR contraindications (e.g., unsafe metal in the body, moderate or severe claustrophobia). All experimental procedures and data sharing were performed according to relevant guidelines and regulations^40^. Participants visited Washington University two different days for magnetic resonance imaging (MRI) scanning and cognitive assessments.

### Gait speed assessment

The four-meter gait test, an established and valid measurement of gait speed, was performed. The test was administered as a part of the motor assessment in the NIH toolbox^41^ which was adapted from the Short Physical Performance Battery^42^. During the gait speed test, participants were instructed to walk four meters at their usual pace. Participants completed one practice session before performing two timed walking tests. Scores were recorded in seconds and the faster performance among the two walks was used as the reported score. Scores were computed in meters per second (m/s). Higher computed scores indicate better gait speed (i.e., fewer seconds to walk four meters).

### Working memory task

An n-back task with 2- and 0-back load levels were used to examine working memory, which was performed within MRI scanner^43^. Blocks of trials consisted of pictures of places, tools, faces and body parts were presented during the test. The four different stimulus types were presented in separate blocks within each run. Within each run, half of the blocks used a 2-back working memory task and half used a 0-back working memory task as a working memory comparison. A 2.5 s cue indicated the task type and target for 0-back at the start of the block. Each of the two runs comprised of eight task blocks (10 trials of 2.5 s each, 25 s in total) and four fixation blocks (15 s each). On each trial, the stimulus was presented for two seconds, followed by a 500 ms inter-task interval^37^. Accuracy across all conditions in the working memory task (%) and median response time for all conditions (RT; ms) were used as variables of interest in the present study. As a measure of working memory, we used 2-back performance as a dependent variable and 0-back performance as a covariate in the following regression analyses.

### Fluid cognition composite score

A comprehensive battery of neuropsychological tests was administered to evaluate cognitive function, including Picture Sequence Memory, Dimensional Change Card Sort, Flanker task, Oral Reading Recognition, Picture Vocabulary, Pattern Comparison, and List Sorting. Specific descriptions regarding the administration and scoring methods of these tests are further detailed in previous studies^44,45^. The subtest scores were averaged to generate composite scores using the NIH Toolbox Cognition Battery. One of the composite scores was the fluid cognition composite score in which executive function test scores (e.g., Flanker, Dimensional Change Card Sort, Picture Sequence Memory, and List Sorting) were converted to normally distributed standard scores, with a mean of 100 and a standard deviation of 15. These standard scores were then averaged to compute the fluid cognition composite score.

### MRI data acquisition

Whole-brain MRI scan was conducted using a customized Siemens (Munich, Germany) 3.0 Tesla Skyra MR scanner at Washington University. A 32-channel head coil (SC72) was used for radio frequency transmission and reception. A high-resolution T1-weighted anatomical image was acquired with gradient echo sequence: field of view = 224 mm, voxel size = 0.7 × 0.7 × 0.7 mm, slice thickness = 0.7 mm, repetition time = 2400 ms, echo time = 2.14 ms, inversion time = 1000 ms, flip angle = 8°, and duration = 7:40 min^37^.

### Cerebellar subregion analysis

The Statistical Parametric Mapping version 12 (Wellcome Department of Cognitive Neurology, London, UK; http://www.fil.ion.ucl.ac.uk) software package’s Spatially Unbiased Infratentorial Template (SUIT) toolbox (version 3.4), running on MATLAB (MathWorks, Natick, MA, version 2018a), was used for cerebellar subregion volume analyses. First, from the T1-weighted high-resolution anatomical images, infratentorial structures (e.g., cerebellum and brainstem) were isolated from the surrounding cortical tissue^46,47^. Cerebellar extraction was visually inspected for proper extraction. Using the unified segmentation procedure, gray matter, white matter, and cerebrospinal fluid were segmented as part of the isolation procedure^48^. Next, gray matter volume segmentations were normalized into the probabilistic SUIT atlas template using the Diffeomorphic Anatomical Registration using Exponentiated Lie algebra (DARTEL) registration approach^49^. Subsequently, cerebellar subregion gray matter volumes (mm^3^) were generated across participants.

### Whole cerebellar and thalamus volume analysis

T1-weighted high-resolution anatomical images were processed using the FreeSurfer (version 5.3.0) automatic cortical reconstruction process for cortical parcellation and subcortical segmentation (recon-all)^50^. Bilateral cerebellar volume and thalamus volume (mm^3^) were calculated from this automated segmentation process.

### Statistical analysis

We used the Shaprio–Wilk test to determine normality of demographic, gait speed, working memory performance, whole cerebellar volume, and cerebellar subregion volumes. First, we investigated the relationship between gait speed and 2-back working memory performance. To accomplish this, we used linear regression models where gait speed was set as an independent variable and working memory performance was included as a dependent variable after controlling for age, sex, years of education, and 0-back working memory performance. Second, we investigated the link between gait speed and cerebellar volumes (bilateral cerebellum and cerebellar subregions). In the linear regression model, gait speed was set as an independent variable and cerebellar subregion volumes were included as dependent variables after controlling for age, sex, years of education, and total intracranial volume (ICV). ICV, estimated by FreeSurfer^50^, was included as a covariate to adjust cerebellar volumes based on previous investigations that identified significant sex-related differences in ICV^51,52^. Third, the relationship between cerebellar subregion volume and 2-back working memory performance was individually explored. Cerebellar volumes were set as independent variables and working memory performance was included as dependent variables after adjusting for age, sex, years of education, 0-back working memory performance, and ICV. All variables except sex (categorical variable) were coded as continuous variables.

Mediation analysis was conducted for the second aim of the study; to examine if cerebellar subregion volume mediated (or partially mediated) the relationship between gait speed and working memory performance. Based on the significant associations that meet the assumption of mediation from the prior linear regression analyses, we conducted a bootstrapped mediation analysis to determine the effect of gait speed (IV; independent variable), indirectly through cerebellar subregion volume (M; mediator), on working memory performance (DV; dependent variable). The mediation analysis package^53^ within SPSS (v. 26.0, IBM, Armonk, NY, USA) was used for mediation analyses. We used the 95% confidence interval obtained from 5000 bootstrap resamples^54^ to explore the indirect mediation effect of cerebellar subregion volume on the relationship between gait speed and working memory performance. As an exploratory analysis, we tested differential relationships between gait speed and cerebellar volumes, and between working memory and cerebellar volumes. For any associations in which there was a significant association between gait speed and cerebellar volume or working memory and cerebellar volume, either gait speed and working memory was further included in the regression model to compare the magnitudes of the bivariate correlations. Age, sex, years of education, and ICV were included as covariates for all analyses.

## Results

### Participants

Among a total of 1206 participants who completed the study protocol, 152 individuals were excluded due to missing data (T1 anatomical data n = 114, data processing error n = 1, and working memory performance n = 37). Thus, 1054 participants’ data were included for the current study. Overall, the participants had an average age of 28.7 years and 14.9 years of education. 54.4% of the participants were women and 74.8% of the sample was white. The average gait speed score of the participants was 1.3 m/s (Table 1).

**Table 1:** Demographics data of the participants.

	Total sample (n = 1054) Mean ± SD
**Demographics**	
Age (years)	28.7 ± 3.6
Female (n, %)	573 (54.4%)
White (n, %)	788 (74.8%)
Education (years)	14.9 ± 1.7
Gait Speed (m/s)	1.3 ± 0.1
**ICV and cerebellum subregion volume (mm**^**3**^**)**	
ICV	1,581,794.7 ± 188,453.0
Bilateral Cerebellum	115,822.8 ± 12,283.4
Bilateral Crus I	38,473.1 ± 938.6
Bilateral VIIb	25,199.7 ± 663.8
Right VIIIa	6970.7 ± 187.8
**Cognitive tests**	
Working memory accuracy (%)	86.9 ± 8.8
Working memory median RT (ms)	862.9 ± 123.7
Fluid cognition composite score	115.3 ± 11.5

SD, standard deviation; ICV, total intracranial volume; RT, response time.

### Association between gait speed and 2-back working memory/fluid cognition composite performance

There was no association between gait speed and 2-back working memory RT (B = − 0.324, *p* = 0.985, 95% CI − 33.66, 33.05., No association between gait speed and 2-back working memory accuracy was detected (B = 1.001, *p* = 0.496, 95% CI − 1.88, 3.88). There was also no significant correlation between gait speed and fluid cognition composite score (B = 3.141, *p* = 0.071, 95% CI − 0.28, 6.56).

### Association between brain volumes and 2-back working memory/fluid cognition composite performance

Bilateral Crus I (B = 0.001, *p* = 0.002, 95% CI 0.0003, 0.002) and bilateral cerebellum (B = 0.0001, *p* = 0.002, 95% CI 0.00003, 0.0001) volumes were positively associated with 2-back working memory accuracy. However, the cerebellar subregion volumes [Bilateral VIIb (B = 0.001, *p* = 0.495, 95% CI − 0.001, 0.002), Right VIIIa (B = 0.003, *p* = 0.070, 95% CI − 0.0002, 0.006), and Bilateral V (B = 0.00008, *p* = 0.925, 95% CI − 0.002, 0.002)], bilateral thalamus volume (B = − 0.0002, *p* = 0.425, 95% CI − 0.0007, 0.0003), and bilateral precentral gyrus thickness (B = − 0.052, *p* = 0.971, 95% CI − 2.94, 2.84) were not associated with 2-back working memory accuracy. None of the cerebellar subregion volumes [Bilateral Crus I (B = − 0.001, *p* = 0.816, 95% CI − 0.008, 0.006), Bilateral VIIb (B = 0.007, *p* = 0.433, 95% CI − 0.01, 0.02), Right VIIIa (B = 0.011, *p* = 0.565, 95% CI − 0.02, 0.04), and Bilateral V (B = − 0.006, *p* = 0.925, 95% CI − 0.002, 0.002)], bilateral cerebellum (B = − 0.0002, *p* = 0.475, 95% CI − 0.001, 0.0004), bilateral thalamus volumes (B = 0.001, *p* = 0.608, 95% CI − 0.005, 0.009), and bilateral precentral gyrus thickness (B = − 11.61, *p* = 0.582, 95% CI − 53.02, 29.79) were associated with 2-back working memory RT.

Next, while greater bilateral Crus I (B = 0.001, *p* = 0.0008, 95% CI 0.0005, 0.001) and right VIIIa (B = 0.003, *p* = 0.034, 95% CI 0.0003, 0.007) volumes were associated with higher fluid cognition composite score, there were no associations between bilateral VIIb (B = 0.001, *p* = 0.215, 95% CI –0.0006, 0.002) bilateral V (B = 0.001, *p* = 0.283, 95% CI − 0.001, 0.003), bilateral cerebellum (B = 0.00005, *p* = 0.156, 95% CI − 0.00002, 0.0001), bilateral thalamus volumes (B = − 0.00006, *p* = 0.828, 95% CI − 0.0006, 0.0005), and bilateral precentral gyrus thickness (B = − 1.52, *p* = 0.353, 95% CI − 4.74, 1.69) and fluid cognition composite score (Table 2).

**Table 2:** Association between cerebellar subregion volumes/gait speed and working memory performance.

	B coefficient	Standardized B coefficient	*p*-value	95% CI for B
**Gait speed**				
2-Back working memory acc	1.001	0.019	0.496	− 1.88, 3.88
2-Back working memory RT	− 0.324	− 0.0004	0.985	− 33.66, 33.05
Fluid cognition composite score	3.141	0.054	0.071	− 0.28, 6.56
**Bilateral Crus I**				
2-Back working memory acc	0.001	0.085	0.002	0.0003, 0.002
2-Back working memory RT	− 0.001	− 0.006	0.816	− 0.008, 0.006
Fluid cognition composite score	0.001	0.100	0.0008	0.0005, 0.001
**Bilateral VIIb**				
2-Back working memory acc	0.001	0.019	0.495	− 0.001, 0.002
2-Back working memory RT	0.007	0.019	0.433	− 0.01, 0.02
Fluid cognition composite score	0.001	0.038	0.215	− 0.0006, 0.002
**Right VIIIa**				
2-Back working memory acc	0.003	0.050	0.070	− 0.0002, 0.006
2-Back working memory RT	0.011	0.014	0.565	− 0.02, 0.04
Fluid cognition composite score	0.003	0.064	0.034	0.0003, 0.007
**Bilateral V**				
2-Back working memory acc	0.00008	0.003	0.925	− 0.002, 0.002
2-Back working memory RT	− 0.006	− 0.013	0.605	− 0.02, 0.01
Fluid cognition composite score	0.001	0.033	0.283	− 0.001, 0.003
**Whole cerebellum**				
2-Back working memory acc	0.0001	0.121	0.002	0.00003, 0.0001
2-Back working memory RT	− 0.0002	− 0.025	0.475	− 0.001, 0.0004
Fluid cognition composite score	0.00005	0.062	0.156	− 0.00002, 0.0001
**Thalamus**				
2-Back working memory acc	− 0.0002	− 0.031	0.425	− 0.0007, 0.0003
2-Back working memory RT	0.001	0.022	0.608	− 0.005, 0.009
Fluid cognition composite score	− 0.00006	− 0.008	0.828	− 0.0006, 0.0005
**Precentral gyrus**				
2-Back working memory acc	− 0.052	− 0.001	0.971	− 2.94, 2.84
2-Back working memory RT	− 11.61	− 0.01	0.582	− 53.02, 29.79
Fluid cognition composite score	− 1.52	− 0.02	0.353	− 4.74, 1.69

The thalamus volume and precentral gyrus thickness were measured as control. 95% CI, 95% confidence interval, PSQI used as a continuous variable; regression analyses were adjusted for age, sex, education year, and ICV for cerebellar subregions; regression analyses were adjusted for age, sex, and education year for gait speed; ACC, accuracy; RT, response time.

### Association between gait speed and cerebellar subregion volumes

Results showed that faster gait speed predicted greater right VIIIa (B = 76.710, *p* = 0.007, 95% CI 20.35, 133.07) and bilateral VIIb (B = 116.207, *p* = 0.045, 95% CI 2.56, 229.84) volumes. However, there was no significant association between gait speed and bilateral Crus I volume (B = 39.713, *p* = 0.783, 95% CI − 244.27, 323.70), bilateral V (B = 81.079, *p* = 0.101, 95% CI − 15.85, 178.01), bilateral cerebellar volume (B = 382.186, *p* = 0.766, 95% CI − 2147.78, 2912.15), bilateral thalamus volumes (B = − 157.835, *p* = 0.390, 95% CI − 517.99, 202.33), and bilateral precentral gyrus thickness (B = 0.016, *p* = 0.580, 95% CI − 0.04, 0.07) (Table 3).

**Table 3:** Association between gait speed and cerebellar subregion volumes.

	B Coefficient	Standardized B Coefficient	*p*-value	95% CI for B
Bilateral crus I	39.7138	0.008	0.783	− 244.27, 323.70
Bilateral VIIb	116.2071	0.060	0.045	2.56, 229.84
Right VIIIa	67.470	0.072	0.019	10.91, 124.03
Bilateral V	81.079	0.049	0.101	− 15.85, 178.01
Whole cerebellum	382.186	0.006	0.766	− 2147.78, 2912.15
Thalamus	− 157.835	− 0.019	0.390	− 517.99, 202.33
Precentral gyrus	0.016	0.017	0.580	− 0.04, 0.07

The thalamus volume and precentral gyrus thickness were measured as control. 95% CI, 95% confidence interval; regression analyses were adjusted for age, sex, education year, and total intracranial volume.

### Exploratory bivariate correlations

Crus I volume was still positively associated with 2-back working memory accuracy (B = 9.889, *p* = 0.001, 95% CI 4.27, 15.50) while there was no significant association between Crus I and gait speed in the regression model (B = 62.972, *p* = 0.662, 95% CI − 219.84, 345.79). Similarly, greater right VIIIa volume was associated with faster gait speed (working memory RT B = 66.322, *p* = 0.021, 95% CI 9.80, 122.83; working memory accuracy B = 67.430, *p* = 0.020, 95% CI 10.81, 124.04), but there were no significant associations between right VIIIa volume and working memory RT (B = 66.232, *p* = 0.074, 95% CI − 6.07, 130.53) and accuracy (B = − 1.755, *p* = 0.964, 95% CI − 78.12, 74.61), respectively. Conversely, the association between bilateral VIIb volume and gait speed became no longer significant (working memory RT B = 70.264, *p* = 0.115, 95% CI − 17.25, 157.78; working memory accuracy B = 72.232, *p* = 0.106, 95% CI − 15.34, 159.80), as did the associations between bilateral VIIb volume and working memory performance (RT B = 58.379, *p* = 0.279, 95% CI − 47.39, 164.15; accuracy B = 37.782, *p* = 0.530, 95% CI − 80.34, 155.90).

## Discussion

We found that (1) faster gait speed is associated with greater bilateral VIIb and right VIIIa volumes; (2) there were no associations between gait speed and 2-back working memory performance; (3) greater bilateral Crus I volume is associated with better 2-back working memory accuracy; (4) there were no hypothesized pathways meeting the requirements to test mediation analysis; and (5) cerebellar subregion volumes were differentially associated with gait speed and working memory such that Crus I volume was associated with working memory accuracy and right VIIIa volume was associated with gait speed.

The present study suggests a selective association of working memory and gait speed to different regions of the cerebellum in young adults. Interestingly, when examining fluid cognition it was found to not only approach a significant relationship with gait speed (*p* = 0.071), but it was also positively related to both greater right VIIIa and Crus I volumes. Lastly, we did not find statistically significant associations between the control brain regions (thalamus volume and precentral gyrus thickness), gait speed, and working memory performance. These control regions were selected based on their role along cerebellar thalamocortical pathways linked to voluntary movement. The lack of any significant association with these control regions suggests a degree of specificity of the present results to the cerebellum.

Prior work investigating the link between working memory and the cerebellum has primarily used verbal working memory^55^, but other modalities employing object and spatial stimuli also elicited cerebellar activations^56^, suggesting cerebellar engagement in non-verbal n-back tasks. The Crus I has been well-recognized as contributors to working memory. For example, working memory-related functional MRI activation during the n-back task was found in the bilateral regions of Crus I^30^. Further, greater cerebellar subregion gray matter volumes including the Crus I predict better working memory performance^28,29^. Collectively, the present results of the positive correlation between cerebellar subregion volumes and n-back task performance align well with the existing body of literature and suggest that the Crus I represent an important component of working memory.

The crucial functions for successfully accomplishing the n-back task encompass encoding, continuous updating of upcoming stimuli, temporary maintenance of stimuli, inhibiting irrelevant stimuli, and directing attention to relevant information^57^. Evidence has been generated concerning a cerebellar role in monitoring expected and observed outcomes and encoding the sequence of stimuli^58^ as well as maintenance of information over temporal delays (i.e., coordination of temporal information)^59^, which are essential components to accomplish working memory tasks^60^. These cerebellar functions may corroborate the association between greater whole cerebellar volume and better working memory performance in the present study. Specifically, the highly-organized circuits between the Crus I and the frontal and parietal cortex constitute a neural network underpinning the central executive mechanism^61,62^. Given that greater gray matter volume is associated with a greater number of dendrites and synapses^63^, greater gray matter volumes may be associated with greater number of synapses or dendrite arborization possibly indicating a better supporting role of the cerebellum interacting with the prefrontal and parietal regions to facilitate successful task accomplishment. This hypothesis, however, remains speculative and further studies should investigate the mechanistic and translational evidence to robustly test this hypothesis.

A previous investigation found the relationship between smaller cerebellar gray matter volume and slower gait speed in older adults^19^. Conversely, we failed to observe a significant association between whole cerebellar gray matter volume and gait speed in younger individuals. However, there were positive associations between VIIIa volume and gait speed. Theories detailing how these cerebellar subregions contribute to motor control have been documented. Human neuroimaging findings suggest the presence of sensorimotor homunculi in the lobule VIII^35^. Further, resting-state functional connectivity evidence has reported that activity in sensorimotor regions correlates with the cerebellar anterior lobe and lobule VIII^64^. This evidence suggests that output from cerebellar lobule VIII targets the premotor cortex^65^, which is involved in motor learning and movement sequences (i.e., gait control) as well as generating signals to the motor cortex (M1) to initiate voluntary movements of walking^19^. Collectively, these results provide evidence that the relationship between the cerebellar volume and gait speed may be specific to the cerebellar subregions of VIIIa in younger individuals.

In community-dwelling older adults, slower baseline gait speed predicts a decline in attention and psychomotor speed over 5 years^7^. Another prospective study reported the relationship between a decline in gait speed and lower attention and executive function performance in older individuals^66^. In support, Montero-Odasso et al. (2012) suggested that gait speed in older adults with cognitive impairment is linked to impaired working memory^67^. The literature concerning the relationship between gait speed and cognitive function has primarily focused on older adults, but the present study extends the existing literature by suggesting that gait speed may not be associated with working memory performance using a large sample of healthy younger adults. Conversely, there was also a marginally significant association between gait speed and fluid cognition composite score (*p* = 0.071). In addition, the right VIIIa volume-gait speed and right VIIIa volume-fluid cognition composite score associations were all statistically significant. This suggests the possibility of a mediating effect of the right VIIIa volume in the associations between gait speed and broader range of executive function tasks, beyond working memory.

### Strengths and limitations

An important strength of the present study was a large and well-characterized younger adult sample (n = 1054). Further, the Human Connectome Project’s high-resolution neuroimaging data were used to elucidate the mediating role of cerebellar subregions in the relationship between gait speed and working memory performance. We only tested selected subregions that may involve both motor and cognitive function (or both) based on previous evidence to test differential associations of regional cerebellar volume with gait speed and working memory. We believe that this hypothesis-driven approach was appropriate to address the research question of the present study as it allowed robust interpretation of the current results. Despite this strength, we may have missed clusters that we would have found if we used a voxel-wise analysis. Therefore, future work should employ a voxel-wise analysis on the cerebellar gray matter maps to better understand about the mediating role of the cerebellum in the association between gait speed and working memory.

Nevertheless, our study is also limited by its cross-sectional design, which warrants caution when interpreting the directionality of the current results. Future longitudinal and interventional studies will be necessary to clarify the role of the cerebellum in the link between gait speed and cognitive function. A further potential limitation of the current study is that the n-back task used in the Human Connectome Project had a non-standard design in that it simultaneously examined category specific representations and working memory, using pictures of places, faces, tools, and body parts. Future studies using standardized n-back tasks need to clarify the relationship between gait speed, working memory, and cerebellar volume found in this study. Further, since the effect size of the reported associations were relatively modest and lacking corrections for multiple comparisons, the present study should be viewed with caution. Lastly, since cortical volumes were not measured in the present study, the association between gait speed, working memory, and cortical volumes should be further investigated in the future.

## Conclusion

In accordance with previous findings, our results suggest that differential associations of regional cerebellar volume with gait speed and working memory. Distinctive connections between the cerebellar subregions, sensorimotor area, and the cerebral cortex may provide the anatomical substrates for the differential contributions of cerebellum to gait speed and working memory. This work also indicates that although there is no association between gait speed and working memory performance, there was a marginally significant relationship between gait speed and fluid cognition composite score. This suggests a possible predictive role of gait speed to composite executive function. This extends the body of literature focusing primarily on older adults, with evidence that the gait speed-cognitive function may differ by age.

## References

[1] Fritz S, Lusardi M (2009). White paper:“Walking speed: the sixth vital sign”. J. Geriatr. Phys. Ther..

[2] Newman AB, Simonsick EM, Naydeck BL, Boudreau RM, Kritchevsky SB, Nevitt MC (2006). Association of long-distance corridor walk performance with mortality, cardiovascular disease, mobility limitation, and disability. JAMA.

[3] Peel NM, Alapatt LJ, Jones LV, Hubbard RE (2019). The association between gait speed and cognitive status in community-dwelling older people: A systematic review and meta-analysis. J. Gerontol. Ser. A..

[4] Demnitz N, Hogan DB, Dawes H, Johansen-Berg H, Ebmeier KP, Poulin MJ (2018). Cognition and mobility show a global association in middle-and late-adulthood: Analyses from the Canadian Longitudinal Study on Aging. Gait Posture..

[5] Taniguchi Y, Yoshida H, Fujiwara Y, Motohashi Y, Shinkai S (2012). A prospective study of gait performance and subsequent cognitive decline in a general population of older Japanese. J. Gerontol. Ser. Biomed. Sci. Med. Sci..

[6] Alfaro-Acha A, Al Snih S, Raji MA, Markides KS, Ottenbacher KJ (2007). Does 8-foot walk time predict cognitive decline in older Mexicans Americans?. J. Am. Geriatr. Soc..

[7] Inzitari M, Newman AB, Yaffe K, Boudreau R, De Rekeneire N, Shorr R (2007). Gait speed predicts decline in attention and psychomotor speed in older adults: The health aging and body composition study. Neuroepidemiology.

[8] Watson NL, Rosano C, Boudreau RM, Simonsick EM, Ferrucci L, Sutton-Tyrrell K (2010). Executive function, memory, and gait speed decline in well-functioning older adults. J. Gerontol. Ser. Biomed. Sci. Med. Sci..

[9] Soumaré A, Tavernier B, Alpérovitch A, Tzourio C, Elbaz A (2009). A cross-sectional and longitudinal study of the relationship between walking speed and cognitive function in community-dwelling elderly people. J. Gerontol. Ser. Biomed. Sci. Med. Sci..

[10] Rasmussen LJH, Caspi A, Ambler A, Broadbent JM, Cohen HJ, d’Arbeloff T (2019). Association of neurocognitive and physical function with gait speed in midlife. JAMA Netw. Open..

[11] Mielke MM, Roberts RO, Savica R, Cha R, Drubach DI, Christianson T (2013). Assessing the temporal relationship between cognition and gait: Slow gait predicts cognitive decline in the Mayo Clinic Study of Aging. J. Gerontol. Ser. Biomed. Sci. Med. Sci..

[12] Abellan van Kan G, Rolland Y, Gillette-Guyonnet S, Gardette V, Annweiler C, Beauchet O (2012). Gait speed, body composition, and dementia. The EPIDOS-Toulouse cohort. J. Gerontol. Ser. Biomed. Sci. Med. Sci..

[13] Holmes G (2007). The Croonian Lectures on the clinical symptoms of cerebellar disease and their interpretation. Lecture II. 1922. Cerebellum Lond Engl..

[14] Nedelescu H, Chowdhury TG, Wable GS, Arbuthnott G, Aoki C (2017). Cerebellar sub-divisions differ in exercise-induced plasticity of noradrenergic axons and in their association with resilience to activity-based anorexia. Brain Struct. Funct..

[15] Dow RS, Moruzzi G (1958). The physiology and pathology of the cerebellum.

[16] Drew T, Andujar J-E, Lajoie K, Yakovenko S (2008). Cortical mechanisms involved in visuomotor coordination during precision walking. Brain Res. Rev..

[17] Mori S, Matsuyama K, Kohyama J, Kobayashi Y, Takakusaki K (1992). Neuronal constituents of postural and locomotor control systems and their interactions in cats. Brain Dev..

[18] Mochizuki H, Ugawa Y (2010). Cerebellar ataxic gait. Brain Nerve Shinkei Kenkyu No Shinpo..

[19] Nadkarni NK, Nunley KA, Aizenstein H, Harris TB, Yaffe K, Satterfield S (2014). Association between cerebellar gray matter volumes, gait speed, and information-processing ability in older adults enrolled in the Health ABC study. J. Gerontol. Ser. Biomed. Sci. Med. Sci..

[20] Callisaya ML, Beare R, Phan TG, Chen J, Srikanth VK (2014). Global and regional associations of smaller cerebral gray and white matter volumes with gait in older people. PLoS One..

[21] Lagarde J, Hantkie O, Hajjioui A, Yelnik A (2009). Neuropsychological disorders induced by cerebellar damage. Ann Phys Rehabil Med..

[22] Ben-Yehudah G, Guediche S, Fiez JA (2007). Cerebellar contributions to verbal working memory: Beyond cognitive theory. The Cerebellum..

[23] Haarmeier T, Thier P (2007). The attentive cerebellum—Myth or reality?. The cerebellum..

[24] Buckner RL, Krienen FM, Castellanos A, Diaz JC, Yeo BT (2011). The organization of the human cerebellum estimated by intrinsic functional connectivity. J. Neurophysiol..

[25] Stoodley CJ (2012). The cerebellum and cognition: Evidence from functional imaging studies. The Cerebellum..

[26] Cabeza R, Nyberg L (2000). Neural bases of learning and memory: Functional neuroimaging evidence. Curr. Opin. Neurol..

[27] Bernard JA, Seidler RD (2013). Relationships between regional cerebellar volume and sensorimotor and cognitive function in young and older adults. The Cerebellum..

[28] Cooper FE, Grube M, Von Kriegstein K, Kumar S, English P, Kelly TP (2012). Distinct critical cerebellar subregions for components of verbal working memory. Neuropsychologia.

[29] Moore DM, D’Mello AM, McGrath LM, Stoodley CJ (2017). The developmental relationship between specific cognitive domains and grey matter in the cerebellum. Dev. Cogn. Neurosci..

[30] Stoodley CJ, Valera EM, Schmahmann JD (2012). Functional topography of the cerebellum for motor and cognitive tasks: an fMRI study. Neuroimage.

[31] Tomasi D, Caparelli EC, Chang L, Ernst T (2005). fMRI-acoustic noise alters brain activation during working memory tasks. Neuroimage.

[32] Eckert MA (2011). Slowing down: Age-related neurobiological predictors of processing speed. Front. Neurosci..

[33] Won J, Faroqi-Shah Y, Callow DD, Williams A, Awoyemi A, Nielson KA, et al. Association between greater cerebellar network connectivity and improved phonemic fluency performance after exercise training in older adults. *The Cerebellum* 1–14 (2021). 10.1007/s12311-020-01218-3 PMC10734642 33507462

[34] Hautzel H, Mottaghy FM, Specht K, Müller H-W, Krause BJ (2009). Evidence of a modality-dependent role of the cerebellum in working memory? An fMRI study comparing verbal and abstract n-back tasks. Neuroimage.

[35] Grodd W, Hülsmann E, Lotze M, Wildgruber D, Erb M (2001). Sensorimotor mapping of the human cerebellum: fMRI evidence of somatotopic organization. Hum. Brain Mapp..

[36] Droby A, El Mendili MM, Giladi N, Hausdorff JM, Maidan I, Mirelman A (2021). Gait and cognitive abnormalities are associated with regional cerebellar atrophy in elderly fallers—A pilot study. Gait Posture..

[37] Van Essen DC, Ugurbil K, Auerbach E, Barch D, Behrens TEJ, Bucholz R (2012). The Human Connectome Project: A data acquisition perspective. Neuroimage.

[38] Dacre J, Colligan M, Clarke T, Ammer JJ, Schiemann J, Chamosa-Pino V (2021). A cerebellar-thalamocortical pathway drives behavioral context-dependent movement initiation. Neuron..

[39] Folstein MF, Folstein SE, McHugh PR (1975). “Mini-mental state”: A practical method for grading the cognitive state of patients for the clinician. J. Psychiatr. Res..

[40] Glasser MF, Smith SM, Marcus DS, Andersson JL, Auerbach EJ, Behrens TE (2016). The human connectome project’s neuroimaging approach. Nat. Neurosci..

[41] Reuben DB, Magasi S, McCreath HE, Bohannon RW, Wang Y-C, Bubela DJ (2013). Motor assessment using the NIH Toolbox. Neurology..

[42] Guralnik JM, Ferrucci L, Pieper CF, Leveille SG, Markides KS, Ostir GV (2000). Lower extremity function and subsequent disability: consistency across studies, predictive models, and value of gait speed alone compared with the short physical performance battery. J. Gerontol. A Biol. Sci. Med. Sci..

[43] WU-Minn HCP. 1200 subjects data release reference manual. https://www.humanconnectome.org (2017).

[44] Gershon RC, Wagster MV, Hendrie HC, Fox NA, Cook KF, Nowinski CJ (2013). NIH toolbox for assessment of neurological and behavioral function. Neurology.

[45] Weintraub S, Dikmen SS, Heaton RK, Tulsky DS, Zelazo PD, Bauer PJ (2013). Cognition assessment using the NIH Toolbox. Neurology.

[46] Diedrichsen J (2006). A spatially unbiased atlas template of the human cerebellum. Neuroimage.

[47] Diedrichsen J, Balsters JH, Flavell J, Cussans E, Ramnani N (2009). A probabilistic MR atlas of the human cerebellum. Neuroimage.

[48] Ashburner J, Friston KJ (2005). Unified segmentation. Neuroimage.

[49] Ashburner J (2007). A fast diffeomorphic image registration algorithm. Neuroimage.

[50] Fischl B (2012). FreeSurfer. Neuroimage.

[51] Raz, N. Aging of the brain and its impact on cognitive performance: integration of structural and functional findings. (2000).

[52] Won J, Alfini AJ, Weiss LR, Nyhuis CC, Spira AP, Callow DD (2019). Caudate volume mediates the interaction between total sleep time and executive function after acute exercise in healthy older adults. Brain Plast..

[53] Hayes AF (2012). PROCESS: A versatile computational tool for observed variable mediation, moderation, and conditional process modeling.

[54] Preacher KJ, Hayes AF (2008). Asymptotic and resampling strategies for assessing and comparing indirect effects in multiple mediator models. Behav. Res Methods..

[55] Chen SA, Desmond JE (2005). Cerebrocerebellar networks during articulatory rehearsal and verbal working memory tasks. Neuroimage.

[56] Hautzel H, Mottaghy FM, Schmidt D, Zemb M, Shah NJ, Müller-Gärtner H-W (2002). Topographic segregation and convergence of verbal, object, shape and spatial working memory in humans. Neurosci. Lett..

[57] Rey-Mermet A, Gade M, Oberauer K (2018). Should we stop thinking about inhibition? Searching for individual and age differences in inhibition ability. J. Exp. Psychol. Learn. Mem. Cogn..

[58] Ackermann H (2008). Cerebellar contributions to speech production and speech perception: psycholinguistic and neurobiological perspectives. Trends Neurosci..

[59] Teki S, Griffiths TD (2016). Brain bases of working memory for time intervals in rhythmic sequences. Front. Neurosci..

[60] Ivry RB, Spencer RM, Zelaznik HN, Diedrichsen J (2002). The cerebellum and event timing. Ann. N. Y. Acad. Sci..

[61] Desmond JE, Chen SA, Shieh PB (2005). Cerebellar transcranial magnetic stimulation impairs verbal working memory. Ann. Neurol. Off. J. Am. Neurol. Assoc. Child Neurol. Soc..

[62] Schumacher EH, Lauber E, Awh E, Jonides J, Smith EE, Koeppe RA (1996). PET evidence for an amodal verbal working memory system. Neuroimage.

[63] Kassem MS, Lagopoulos J, Stait-Gardner T, Price WS, Chohan TW, Arnold JC (2013). Stress-induced grey matter loss determined by MRI is primarily due to loss of dendrites and their synapses. Mol. Neurobiol..

[64] O’reilly JX, Beckmann CF, Tomassini V, Ramnani N, Johansen-Berg H (2010). Distinct and overlapping functional zones in the cerebellum defined by resting state functional connectivity. Cereb. Cortex..

[65] Hashimoto M, Takahara D, Hirata Y, Inoue K, Miyachi S, Nambu A (2010). Motor and non-motor projections from the cerebellum to rostrocaudally distinct sectors of the dorsal premotor cortex in macaques. Eur. J. Neurosci..

[66] Holtzer R, Wang C, Lipton R, Verghese J (2012). The protective effects of executive functions and episodic memory on gait speed decline in aging defined in the context of cognitive reserve. J. Am. Geriatr. Soc..

[67] Montero-Odasso M, Verghese J, Beauchet O, Hausdorff JM (2012). Gait and cognition: a complementary approach to understanding brain function and the risk of falling. J. Am. Geriatr. Soc..

